# Towards an Artificially Empathic Conversational Agent for Mental Health Applications: System Design and User Perceptions

**DOI:** 10.2196/10148

**Published:** 2018-06-26

**Authors:** Robert R Morris, Kareem Kouddous, Rohan Kshirsagar, Stephen M Schueller

**Affiliations:** ^1^ Koko New York, NY United States; ^2^ Department of Computer Science Columbia University New York, NY United States; ^3^ Center for Behavioral Intervention Technologies Department of Preventive Medicine, Feinberg School of Medicine Northwestern University Chicago, IL United States

**Keywords:** conversational agents, mental health, empathy, crowdsourcing, peer support

## Abstract

**Background:**

Conversational agents cannot yet express empathy in nuanced ways that account for the unique circumstances of the user. Agents that possess this faculty could be used to enhance digital mental health interventions.

**Objective:**

We sought to design a conversational agent that could express empathic support in ways that might approach, or even match, human capabilities. Another aim was to assess how users might appraise such a system.

**Methods:**

Our system used a corpus-based approach to simulate expressed empathy. Responses from an existing pool of online peer support data were repurposed by the agent and presented to the user. Information retrieval techniques and word embeddings were used to select historical responses that best matched a user’s concerns. We collected ratings from 37,169 users to evaluate the system. Additionally, we conducted a controlled experiment (N=1284) to test whether the alleged source of a response (human or machine) might change user perceptions.

**Results:**

The majority of responses created by the agent (2986/3770, 79.20%) were deemed acceptable by users. However, users significantly preferred the efforts of their peers (*P*<.001). This effect was maintained in a controlled study (*P*=.02), even when the only difference in responses was whether they were framed as coming from a human or a machine.

**Conclusions:**

Our system illustrates a novel way for machines to construct nuanced and personalized empathic utterances. However, the design had significant limitations and further research is needed to make this approach viable. Our controlled study suggests that even in ideal conditions, nonhuman agents may struggle to express empathy as well as humans. The ethical implications of empathic agents, as well as their potential iatrogenic effects, are also discussed.

## Introduction

### Background

Conversational agents are software applications that respond to users with natural language, often with the goal of helping a user complete a task [1]. These agents reduce the need for visual interfaces or input devices and allow for more seamless interaction between humans and machines. Such tools have evolved rapidly in recent years, from smartphone integrations (such as Apple’s Siri, Android’s Google Now, Samsung’s S Voice, and Microsoft’s Cortana) to stand-alone devices that are placed within the home (Amazon’s Alexa and Google Home).

While ostensibly designed for utilitarian purposes–such as booking an airline ticket, ordering food, or playing music–some of these agents are also programmed to react to the user’s emotional state. For instance, if you say you’re feeling sad, Siri might reply, “I’m sorry to hear that” or, “from our deepest sadness springs our deepest joy.” Unfortunately, as of today, Siri is quick to repeat herself and she can only draw from a limited set of simple, generic phrases and platitudes. Furthermore, some of her remarks could be interpreted as flippant and insensitive (eg, “I would give you a shoulder to cry on, if I had one.”). If you offer a more detailed account of a stressful situation (eg, “I’m really nervous about an upcoming exam, I think I’m going to fail.”), she’ll simply say that she doesn’t understand. Alexa and Google Home exhibit similar characteristics; they occasionally acknowledge the distress of the user and react in kind, but only in a very general sense. In short, their ability to empathize remains inchoate.

This issue might be forgivable for some agents, such as those that are designed mostly for transactional interactions, but it becomes problematic when automated systems are increasingly being called upon to support health care applications, especially in the domain of mental health. Indeed, many new mental health applications in the market are making use of conversational agents and text-based dialogue systems [2]. For example, commercially available products like Woebot, 7Cups, and Koko have used chatbots for various tasks, such as providing psychological assessments and psychoeducational materials. These agents can be deployed on messenger systems (eg, Facebook Messenger, Kik, Twitter) and are designed to present mental health materials in an interactive and conversational style.

Woebot, a mental health app that relies exclusively on a bot for user interactions, was evaluated in a two-week trial among college students who self-identified as experiencing depression and anxiety [3]. Participants who used the service experienced a significant reduction in symptoms of depression whereas those who received an information control did not [3]. Similarly, “Shim” is a mental health chatbot that was designed for a nonclinical sample. In a controlled trial, users who engaged with the Shim bot experienced increased well-being and reduced perceived stress [4]. Thus, it appears that it is feasible to build such automated conversational agents, they can be engaging enough that some people may continue to use them over a relatively short period of time (ie, up to two weeks), and that those who use them report benefits on a variety of metrics related to mental health, including well-being, stress, and depression.

Although these mental health agents frequently ask users to disclose personal anxieties and vulnerabilities, their reactions remain limited to simple, short remarks. As of today, when a user discloses negative thoughts to Woebot, the agent says, “I see, you certainly have a lot going on at the moment.” The bot on Koko does not provide any sort of empathic reaction and simply acknowledges that the message has been received (“Thanks for sharing… sending this off to the Koko community”). As with Alexa and Siri, these agents are currently unable to reflect any deep understanding of the user’s particular situation.

This simple approach may be sensible today, given the current state of these technologies. An attempt to show empathy that misses the mark, however slightly, could offend the user. However, agents with sophisticated empathic abilities (ie, agents that seem to truly understand the user’s emotional experience) could have a profound effect on the user. Previous research suggests that this ability can lead to increased user satisfaction [5] and affinity for the agent [6].

Regarding agents in mental health applications specifically, strong empathic abilities could be especially useful. Empathy is a commonly cited “nonspecific” factor in psychotherapy that has been proposed to be a major driver of the benefits that clients derive from treatment [7]. Empathy is a strong predictor of therapeutic alliance, which has itself been found to predict outcomes in various trials of psychotherapy [8]. In client-centered therapy, therapists might convey empathy through techniques such as active listening, reflection, and adopting a nonjudgmental stance and warm tone. Conversational agents might be able to model similar behaviors, at least through speech.

Researchers have taken strides in this direction, exploring ways for software agents to mimic emotions [9,10], adjust personality style [11], and programmatically communicate expressions of concern [12]. Yet, more work is needed before these agents can pass anything resembling an *empathic* Turing test—that is, the ability to engage in empathic dialogue in ways that are indistinguishable from a real human. To achieve this goal, the agent may need to express shared understanding, offer new perspectives, and generally take into account the unique situation and feelings experienced by the user. In the words of Carl Rogers, a pioneer of person-centered psychotherapy:

Being empathic is to perceive the internal frame of reference of another with accuracy and with the emotional components and meanings which pertain thereto... it means to sense the hurt or the pleasure of another as he senses it and to perceive the causes thereof as he perceives them.

13

Considerable advances in commonsense reasoning and natural language processing would be needed to generate this kind of empathy from scratch. In this paper, we explored an alternative approach that sidestepped many of the remaining, unsolved challenges for artificial intelligence. Specifically, we examined whether a conversational agent could express rich, empathic understanding simply by repurposing preexisting emotional support data.

### Goal of This Work

The goal of this work was to take initial steps towards building a conversational agent that can respond immediately, and convincingly, with empathic verisimilitude. To do this, we used a corpus-based approach; preexisting emotional support statements were drawn from a large corpus of online interactions and were presented as if they were authored by the agent, creating the semblance of personalized, empathic expression. We used information retrieval techniques and word embeddings to automate this process in real-time.

We first conducted a preliminary test of this system, assessing performance metrics and user perceptions. In a separate controlled study, we examined the upper limit of such a system; that is, if we are able to simulate an empathic agent that performs at the level of a human peer, how is it perceived by users? Lastly, we discuss some of the ethical implications of these types of systems and some of the unintended consequences that might attend them.

### System Design

#### Koko Platform

Emotional support statements were drawn from a corpus of Koko data. Koko is a mobile, peer-to-peer platform that aims to promote emotional resilience [14] that was derived from *Panoply*, a Web-based platform that was previously shown to reduce symptoms of depression [15]. Koko employs a text-based user-interface and is available on various messaging platforms (eg, Kik, Facebook Messenger, Twitter), as well as mobile and desktop browsers. A chatbot (KokoBot) is used to introduce users to the platform, teach cognitive reappraisal skills, and facilitate peer-to-peer interactions.

Unlike some other peer support platforms, Koko does not support repeated interactions between users (such as extended, private messages or multithreaded comments). Instead, all interactions follow a simple *post-response* format, with the chatbot passing messages between users seeking help and those who have opted to give help. When posting on Koko, users are prompted to describe a stressful situation and record any associated negative thoughts they may have. Users are taught to respond to each other’s posts with positive reappraisals and messages of acceptance.

Character count limits of 950 and 600 are imposed on posts and responses, respectively. This convention further distinguishes Koko from many online peer support forums, such as those found on Reddit or Facebook. Most support forums do not impose character count limits, allowing users to write as much as they like. Long-form posts would be very difficult to repurpose for the short, query-response interactions one typically has with a conversational agent. By contrast, posts and responses on Koko are generally quite short, containing an average of 223.05 (SD 159.42) and 222.65 characters (SD 136.35), respectively.

Users rate the quality of each response they receive on a single-item, three-point Likert scale (*good*, *ok*, *bad*). For pragmatic reasons, we were not able to conduct multi-item assessments, such as those intended to capture various facets of *perceived empathy*. Lengthier assessments such as these are difficult to employ in consumer applications, since they can lead to high levels of user attrition. Additional outcome measures would need to be collected to specifically examine whether users found the responses empathic in the way this construct is traditionally operationalized. That being said, it is important to note that users on Koko are explicitly told to help each other feel supported and understood. As such, it is likely that many users considered various empathic factors when making their ratings (eg, *Did this response make me feel understood? Did it make me feel better?*). Future studies should deploy longer assessments to help test this issue more systematically.

All peer interactions on Koko were supervised by a hybrid human-machine moderation system. A suite of deep neural nets was used to detect abusive behavior, inappropriate contributions, users in crisis, and various other infractions (see Kshirsagar et al [16] for a specific description of our crisis model and Calvo et al [17] for a general discussion of how natural language processing can be used to glean mental health information from text). Users at risk of harming themselves were referred to crisis resources, while those who behaved maliciously were banned from the platform.

#### Corpus

The corpus of peer interactions consisted of 72,785 posts and 339,983 responses. Content that was quarantined by our moderation system was not included, nor were responses that received “bad” ratings by users. Additionally, we removed any responses that included solicitations to chat on other platforms, such as Kik or Instagram. All of the aforementioned properties of the Koko platform (the style of interaction, the character limits, the strict moderation procedures, and the user labels) made for a highly structured corpus of data. Without this structure, it would have been very difficult to undertake the information retrieval approach we pursued. The anonymous nature of the dataset is also important, as it excludes cases where interaction partners addressed each other by name or drew upon shared experiences.

#### Back-End System

The two components of this system were: (1) a back-end system to automatically pair previously archived responses with incoming posts, and (2) a front-end system to display responses and solicit user feedback. We used an information retrieval approach to automatically return responses to the user. For any incoming post on our network, we searched for similar posts within the existing corpus of peer support interactions on Koko. Once we found a post, we looked at the associated responses. If we found one that was rated favorably, we returned it to the user (see Table 1 for examples).

The success of this method depends on the similarity between the incoming post and any that were archived previously. In an idealized case, when an incoming post matches a historical post verbatim, there is no need to solicit new responses from the network; responses to the historical post should be very well-matched to the new, incoming post. Conversely, if two posts are highly dissimilar, responses to one would not necessarily apply to the other.

To compute similarity between posts, we first used Elasticsearch [18], an open source full-text search engine that is designed to be faster and more scalable than typical relational databases. Specifically, we used Elasticsearch’s *more like this* query, which computes the terms in a document with the highest term frequency-inverse document frequency (*tf-idf)* weights. The *tf-idf* ratio identifies sets of terms that are most discriminative for a document (in our case a “post”) [19]. The top such terms are then used in a disjunctive query to find documents containing similar terms. This approach was used to return a set of potentially relevant posts as quickly as possible, so the system could respond in near real-time.

**Table 1:** Examples from the full end-to-end system. Spelling and grammatical errors, as well as profanities, are preserved to illustrate the nature of the peer interactions on the network. Here, we illustrate examples of responses that were deemed *good*, *ok*, and *bad*. The response that was rated *bad* (depicted in row 3) exhibited a mismatch in gender pronouns.

Incoming Post	Retrieved Post	Retrieved Response	User Rating
I have really bad social anxiety and im really afraid of public places, i cant go to the grocery store alone and im 17. i'll never get over it	I had really bad anxiety and I'm always scared to go places or to get out the house. And my mom doesn't do anything about it I'll never get over anxiety	I deal with anxiety too but it will get better. I know it seems hard right now but it will get better, please never forget that. I know it seems really hard but life gets better. Never forget that no matter what happens you can always get through it. It'll be okay. It will.	Good
I'm anxious about a class I'm taking because I don't know anyone in it. The class is going to be tricky because I skipped two levels to take this third level of the class, and it feels like i don't have anyone that can help me. :-( My anxiousness is going to get the best of me and cause me to fail the class by not participating.	It's the second week of school, and I'm very stressed about the amount of homework that I have and the level of classes I chose to take. I don't know how to handle it well that I'm going to fail all my classes and I'm so lost I don't think it will get better	I definitely understand how stressful that can be, and I'm sorry you're feeling this way An important thing to remember is that everyone is different. Sadly too many often place too much pressure on students, but just remember YOU and your health come FIRST.	OK
This Girl i like likes me too but i think ill fuck things up cause i am not really confident. Im really not confident	I really like this guy and he used to be really awkward around me and I was with him too but all of the sudden he's gotten really smooth and confident but I still can't even look him in the eye without my fave turning so red I just don't think I can be confident I really want to but I don't know how	hey, this is sweet. and I myself am a guy and its best to be yourself around us guys, that's what we love. a girl who is herself! good luck!	Bad

Among the set of posts that were returned by the *more like this* query, we then computed a word mover’s distance (WMD) score [20] to further refine our measure of similarity between the incoming post and those returned by Elasticsearch. The WMD approach uses *word2vec*, a word-embedding procedure that learns a vector representation of words [21]. The word vectors encode the semantic relationships between words, positioning related words in nearby vector space. We used Google’s pretrained *word2vec* model which includes word vectors for 3 million words and phrases, trained on approximately 100 billion words from the Google News dataset. WMD uses the *word2vec* embeddings to compute the distance between two documents, finding semantic similarities even when the documents have few words in common.

The WMD score was used to not only rank documents, but also to set a threshold to help decide whether the similarity between posts was sufficient to recycle a previous reply. We set a threshold that would return matches for approximately 10% of posts. Having no data to start with, we were not sure how well the documents would match in practice and what level of document similarity would lead to favorable outcomes. We reasoned that this threshold would at least give us a reasonably large dataset of user ratings to help evaluate the system and refine it further. Extremes on either end would be impractical; responding to 100% of posts would yield an intolerable false positive rate, whereas responding to 1% would likely improve precision, but not yield as much user feedback.

#### Front-End User Experience

**Figure 1:**
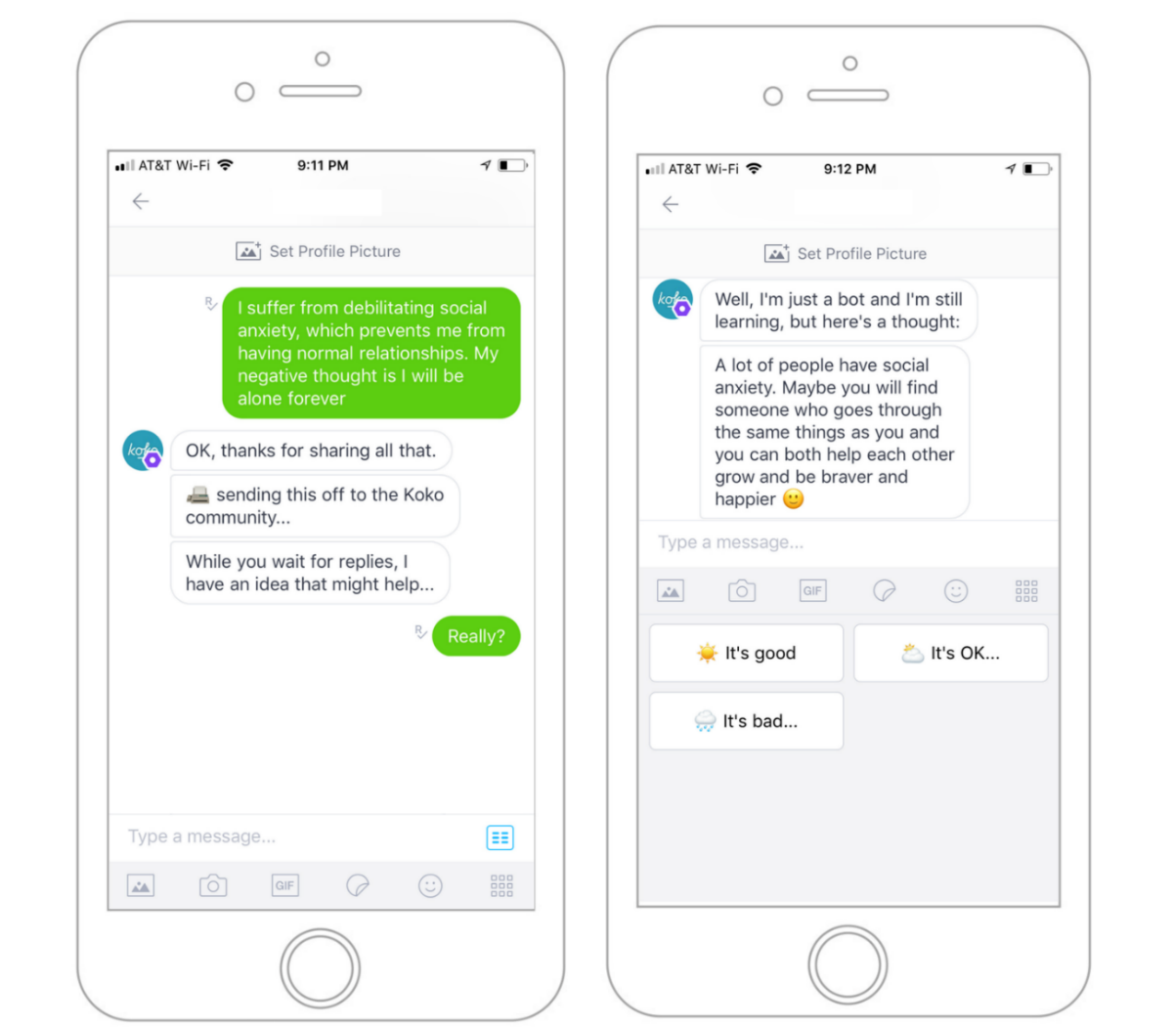
A screenshot of the Koko platform, as seen on the Kik Messenger service. The agent automatically retrieves a response for the user while peer responses are collected from the network.

Once a preexisting response was retrieved, it was presented to the user as though it was algorithmically generated by the robot. Users were not told that the agent was passing off other people’s words as its own. Immediately after posting, the chatbot informed users that it might have a response of its own. Specifically, the bot said, “While you wait for responses, I may have an idea that might help…” After reading the response, users were asked to rate it on a three-point Likert scale (*good*, *ok*, *bad*), as shown in Figure 1. Users were encouraged to be honest and were told that their ratings would be hidden.

#### Participants

Participants included 37,169 individuals who signed up for Koko between mid-August and mid-September of 2016. These users were not asked to reveal their age, gender, or other personal information, so the demographics of this sample are unknown. However, in a separate survey of Koko users from 2017 (N=496), 65% identified as female and the majority were young adults (mean 18.24, SD 5.80). No significant changes occurred in the Koko platform or advertising methods; thus, it is likely that users in 2016 had similar characteristics as those surveyed in 2017. As with other research on other commercial well-being apps [22], Koko users accept a user agreement outlining the privacy policies and how usage data might be used for research purposes. The terms and conditions note that, “we do not require you to provide any personal information” and that, “we may share some or all of this usage with third parties in connection with research, analytics, or similar purposes.” Users are told that their usage data may be used, “in aggregate form, that is, as a statistical measure, but not in a manner that would identify you personally.”

#### Results

**Figure 2:**
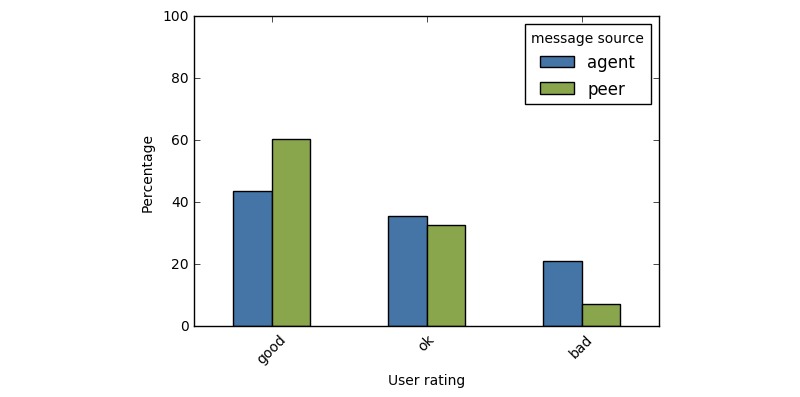
Distribution of user ratings for responses that were created by the nonhuman agent or human peers.

All retrieved responses were located and presented to the user within one second. The actual latency was likely lower but the timestamps on the data we evaluated only resolved to the nearest second. We evaluated user ratings for responses that came from the agent and from peers. In the time period of our analysis, we collected 3770 responses from the bot and 43,596 from peers. Response ratings differed significantly across conditions (see Figure 2). Responses composed by peers were significantly more likely to be rated as good compared to responses that came from the agent (χ^2^(2)=981.20, *P*<.001). However, 79.20% (2986/3770) of responses from the system were deemed *ok* or *good*, suggesting users mostly found the results acceptable (see Figure 2).

#### Conclusions

To automatically retrieve responses, we employed an unsupervised learning method. This approach, while useful to bootstrap the system and collect user feedback, was not sufficient on its own. Responses from actual peers were rated significantly higher than responses generated from the automated system.

Inspection of the data revealed some serious errors due to gender discrepancies. For example, a match between posts might be incredibly close in all respects, except that the subject of one is a female while the other is a male; this led to situations in which a male user was inadvertently assumed to be a female (see row 3 in Table 1). This issue illustrates how a single phrase or word could make two posts quite dissimilar semantically even when much of the raw content overlaps considerably. The system also struggled when it retrieved responses that contained first-person personal accounts. Some of these accounts should not have been repurposed by the artificial agent (eg, “I’ve also struggled with an eating disorder...”).

However, despite these shortcomings, it is perhaps surprising that the majority of responses from the bot were rated favorably. Even with a very simple, unsupervised model, many of the responses generated by the system were well-received. It is also worth noting that the bot was fairly ambitious in its attempts to empathize with the users; these were nuanced responses that addressed specific elements of the poster’s concerns and so there was a great deal of potential for error.

Fortunately, there are many ways this system could be improved upon. In the future, other features could be added to the model to better capture the similarity between incoming and previously archived posts. For example, measures of linguistic style, sentiment, and topic could be included as additional features. Furthermore, the outcome ratings from users could be used to help train supervised models. These models would likely improve as the training set increases in size. As of this writing, there has been a more than tenfold increase in the size of the corpus. As more peers interact with the system, and more outcome data is collected, the models could grow increasingly accurate.

Still, even with larger datasets and improved models, the upper bound of this system remains an open question. When it comes to expressed empathy, people may always prefer humans to agents. To explore this question directly, we conducted a controlled study on the Koko platform that randomly attributed peer responses to either an agent or other humans.

## Methods

Participants included 1284 Koko users who joined the platform between January 18 and 23 in 2018. At sign up, a segment of incoming Koko users was randomly assigned to one of two conditions. In the control condition, users were shown responses from their peers as usual. In the experimental condition, users also received peer responses, but they were told they had come from an artificial agent. To limit the potential impact of this experiment on the greater Koko community, we randomly allotted 2/3 of users to the control condition and 1/3 to the experimental condition, as opposed to a 50/50 random split.

The only part of the experience that differed between conditions was the notification that preceded the delivery of the peer response. In the control condition, before returning a response, the bot said, “Someone replied to your post. Let’s check it out.” In the experimental condition, before returning a response, the bot said, “While you wait for responses, maybe I can help… I'm just a robot and I'm still learning, but here's a thought.” The language was intentionally equivocal and submissive, in the hopes that the user would be more likely to forgive the system, should it fail. This approach also served to lower expectations, an approach that can mitigate disappointment when users experience a robot’s shortcomings [23]. Users in both conditions rated it on the same three-point scale that was used previously. The manipulation was only performed the first time a user received a response, therefore ratings for subsequent responses were not included in the analysis.

## Results

### Findings

**Figure 3:**
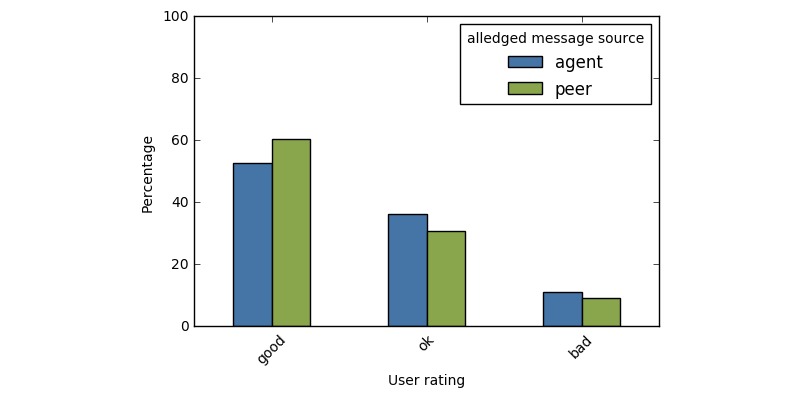
Distribution of ratings when users were told responses came from a peer, as compared to an artificial agent.

Users rated responses less favorably when they were told they came from an agent compared to a peer (see Figure 3). A chi-square analysis revealed a significant difference in the distribution of ratings between the two groups (χ^2^(2)=9.23, *P*<.01). When users believed an agent composed the response, they were significantly less likely to review it favorably, with only 51.6% (227/440) of users rating the response as *good* compared to 60.6% (510/842) of users rating responses as *good* when told the response came from a peer.

### Conclusions

Despite the fact that all responses in this experiment were written by peers, and therefore should not differ in quality, the alleged source of these responses led to different perceptions.

This data is preliminary and may be specific to the particular context in which it was collected. However, it is clear that users harbored some resistance to the agent’s empathic overtures. Future research is needed to better understand what the source of this resistance could be.

## Discussion

### Principal Findings

We created a new method through which conversational agents might simulate empathic expression. Specifically, we explored ways in which preexisting peer support data could be repurposed to help nonhuman agents express empathy. During the time in which it was deployed, the majority of the system’s responses were well received by users. However, responses that were created by actual peers were deemed significantly more favorable. Better models and additional data could improve the performance of such a system, but an open question remains as to whether nonhuman agents could ever achieve parity with humans on empathic expression.

To explore this latter question, we conducted an experiment to see how the framing of an empathic message might affect how it is perceived. Users rated responses less favorably when they were led to believe they came from a nonhuman agent. This suggests that when it comes to empathic expression, conversational agents might be at a perpetual disadvantage. This is interesting given that the conceptual basis for human and machine comparisons often falls back to the Turing test or the degree to which a machine’s behavior would be indistinguishable from that of a human. Our findings suggest that machines might have to do even better than humans to be considered at the same point of emotional intelligence with empathic expressions.

One unique aspect of our approach was to create a system powered by a corpus of peer-created content. This approach differs from many technological tools that digitize skill-training approaches through didactic modules or interactive features or allow for peer communication through unstructured synchronous (eg, chatrooms or chat platforms) or asynchronous (eg, forums) methods. As these applications essentially digitize previous forms of interaction, many have adapted traditional techniques into either visually-driven technologies such as apps [24], or created chatbots that provide education or instruction in these skills themselves [3,4,14]. Such an approach takes advantage of the years of advances in clinical understanding and science of behavior change but fails to capitalize on the full affordances of new technologies. That is, such technologies allow space, time, and availability of a provider to be transcended but do not significantly revolutionize the delivery of services. Allowing peers to contribute to a platform and then scaling up automation on the basis of their contributions truly moves towards the democratization of delivery and contribution of services. It has been noted elsewhere that health care relies too heavily on “consumable interventions,” which are interventions that once used, can never be used again [25]. Technologies are generally nonconsumable interventions in the sense that they can be used repeatedly without exhausting their therapeutic power to help additional people. Peer-powered interventions go a step further; that is, each use of the platform can actually contribute additional benefit to all future users.

Furthermore, other uses of large corpora of empathic chats have demonstrated that such data can provide important contributions to the understanding of actionable strategies to enhance crisis counseling [26] and therapist-client interactions [27]. Such understanding could contribute to the design of automated systems (as was done in this study) and could be applied to human-only interactions (ie, counseling or psychotherapy), or could promote development of human-machine combinations of supportive tools. Elsewhere the exploration of human-machine combinations based on principles of empathic conversations and cognitive restructuring has been found to be a helpful step in allowing peers to have supportive conversations that mirror some processes in psychotherapeutic interactions [28]. Large corpora, applications of data science, and thoughtful design will likely all increase the quality of empathic expressions in the technologies of the future.

### Limitations

There were several limitations to how the system was evaluated and designed. For example, the full end-to-end system was not tested in a controlled study with random assignment. As such, we do not know how our system affected other user behaviors, such as retention and overall engagement with the platform. The system design could also be improved upon. We found some success using unsupervised methods from information retrieval, but more complex models could be employed in the future.

With stronger models, the system could be more confident about when to draw upon preexisting responses and when to use some other approach. Hybrid approaches that combine corpus-based techniques with speech synthesis models could help provide more overall coverage for the system and could be used to create novel utterances that might even outperform those of a peer.

The controlled study had several shortcomings as well, as it was conducted within a very specific context (the Koko application). A similar study should be conducted with various agents within various applications to see if the effect generalizes across settings. Furthermore, we were not able to follow-up with users to assess the credibility of our experimental manipulation. It is possible that users were not fully convinced they were interacting with a machine, despite us claiming otherwise.

Lastly, it is possible the bot’s personality and language style influenced the findings. The way the system framed its efforts could have had a huge effect on how its responses were received. In particular, the bot’s self-deprecatory stance may have affected user perceptions. Guided by past research [23], we employed this approach in the hope that it might make users more lenient in their judgments of the bot. However, it is also possible that this approach led users to scrutinize the agent more closely and appraise its contributions more critically. Furthermore, past research suggests different agent personalities can have differential effects on how they are perceived [29,30], and so the overall personality of the Koko bot may have also affected user perceptions. Future research is needed to better assess the potential impact of these variables.

### Conclusions

Despite its limitations, our approach suggests that it may be possible to simulate empathic expressions by drawing upon a rich corpus of social support data. Theoretically, it might eventually be possible to build a system that draws from millions of candidate responses with near perfect precision. The right response for the right user at the right time could automatically be selected, edited, and returned by the system. This capacity could help agents better serve users within the context of mental health interventions.

Despite these advances, there remains a real question as to whether such a system, even one as idealized as this, could ever outperform a peer. Nonhuman agents do not have lived human experience and so their attempts to express empathy might always appear inauthentic. The effort required to compose a supportive response may be as important as its actual substance. It can be powerful to know that someone else has listened to you, thought deeply about your situation, and then took the time to craft a considerate response. All of this is lost when a machine algorithm automatically generates a response. People often attribute human characteristics to machines and treat them similarly as real people [31], but an agent’s empathic expressions may always be perceived slightly differently than a human’s. It is possible that expressed empathy is a domain in which it will always be hard, if not impossible, for a robot to match human levels of performance.

Even if agents only provide, at most, a simulacrum of empathy, there are still important ethical implications to consider. In this paper, we examined some of the immediate, short-term effects of artificial empathic expression. Future work should assess how humans might relate to empathic agents over longer time periods, especially within the context of mental health interventions. The potential effects on well-being and social relationships are not well understood and many questions remain. Would people eventually seek emotional support from machines, rather than their friends and family? What might a machine miss in the course of these interactions? How can we get the benefits of empathic machines, without sacrificing existing human-human relationships, or otherwise imperiling the user? As Sherry Turkle warns, there is a risk that a robot that, “begins as a solution ends up a usurper” [32]. The Institute of Electrical and Electronics Engineers (IEEE) report on Ethically Aligned Design outlined similar concerns, arguing that agents that express emotion may have unintended effects on the user, such as over-bonding and misplaced trust [33]. To avoid these pitfalls, while still maximizing the potential benefits of this technology, designers will need to carefully scrutinize how these systems affect the well-being of users over time.

## Abbreviations

IEEE: Institute of Electrical and Electronics Engineers
Tf-idf: term frequency-inverse document frequency
WMD: word mover’s distance
